# Electrical and Thermal Transport Properties of Layered Superconducting Ca_10_(Pt_4_As_8_)((Fe_0.86_Pt_0.14_)_2_As_2_)_5_ Single Crystal

**DOI:** 10.3390/ma12030474

**Published:** 2019-02-04

**Authors:** Dapeng Wu, Xiaodong Meng, Yingying Zhai, Huaming Yu, Jiao Yu, Yang Qi

**Affiliations:** 1 College of Science, Liaoning Shihua University, Fushun 113001, China; mengxioadong98@gmail.com (X.M.); h.m.yu@163.com (H.Y.); yujiaojoy@hotmail.com (J.Y.); 2 College of Computer Science and Engineering, Northeastern University, Shenyang 110004, China; zyy@cc.neu.edu.cn; 3 Institute of Materials Physics and Chemistry, School of Materials Science and Engineering, Northeastern University, Shenyang 110004, China; qiyang1960@163.com

**Keywords:** electronic anisotropy, coherence length, upper critical field, Hall effect, thermal conductivity

## Abstract

We have synthesized single crystals of iron-based superconducting Ca_10_(Pt_4_As_8_)((Fe_0.86_Pt_0.14_)_2_As_2_)_5_ and performed extensive measurements on their transport properties. A remarkable difference in the behavior and a large anisotropy between in-plane and out-of-plane resistivity was observed. Disorder could explain the in-plane square-root temperature dependence resistivity, and interlayer incoherent scattering may contribute to the out-of-plane transport property. Along the ab plane, the estimated value of the coherence length is 15.5 Å. From measurements of the upper critical magnetic field *H_c2_* (*T* ≥ 20 K), we estimate *H_c2_*(0) = 313 T. Thermal conductivity for Ca_10_(Pt_4_As_8_)((Fe_0.86_Pt_0.14_)_2_As_2_)_5_ is relatively small, which can be accounted for by the disorder in the crystal and the low-charge carrier density as verified by the Hall effect.

## 1. Introduction

**Figure 1:**
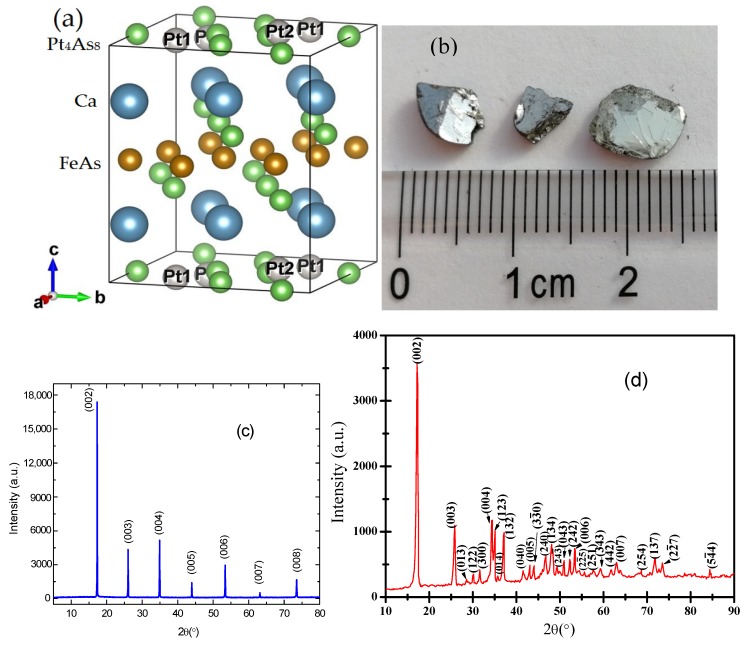
(**a**) Crystal structure of Ca_10_Pt_4_As_8_(Fe_2_As_2_)_5_; (**b**) as prepared Ca_10_(Pt_4_As_8_)(Fe_2−x_Pt_x_As_2_)_5_ single crystals; (**c**) XRD spectrum with index peaks of Ca_10_(Pt_4_As_8_)(Fe_2−x_Pt_x_As_2_)_5_ measured on single crystals along the c-axis at room temperature; (**d**) XRD spectrum of powder Ca_10_(Pt_4_As_8_)(Fe_2−x_Pt_x_As_2_)_5_ at room temperature.

The Ca_10_(Pt*_n_*As_8_)(Fe_2_As_2_)_5_ (n = 3 or 4) system was first reported to exhibit superconductivity with a wide range of transition temperatures in 2011 [1,2,3]. After that, many studies have appeared in the past seven years [4,5,6,7,8]. Ca_10_(Pt_4_As_8_)(Fe_2_As_2_)_5_ has a layered structure consisting of superconducting FeAs layers separated by the spacer layers arranged as Ca–Pt_4_As_8_–Ca, which is shown in Figure 1a. This superconductor has been reported to crystallize in possible space groups including *P*4/n (tetragonal) [2,3], *P*2_1_/n (monoclinic) [7], and $P\bar{1}$ (triclinic) [1,3]. The structure of the Pt_4_As_8_ spacer layer is similar to a square lattice of As atoms, one fifth of the As atoms are replaced by substitutional Pt1 atoms [2], and the same amount of Pt2 atoms are interstitial, which leads to the displacement of As atoms from their ideal positions to form As dimers [2]. Because of the constraint from the As–As dimers and the FeAs sublattices, substitutional Pt1 atoms can sit in plane while the interstitial Pt2 atoms sit on the site either above or below the plane against Ca ions.

Most FeAs-based superconductors need doping to induce the superconductivity; Ca_10_(Pt_4_As_8_)(Fe_2_As_2_)_5_ allows doping on the Ca site or on the Fe site. In particular, Pt doping on the Fe site could bring faults and disorder into the crystal and also adjust the *T*_c_ [2,5]. By studying its structure and physical properties, the origin of superconductivity may be elucidated. The interlayer distance of Ca_10_(Pt_4_As_8_)(Fe_2_As_2_)_5_ is reported to be ~10.5 Å [2,3], which is larger than many other FeAs-based superconductors, and thus its properties are expected to be anisotropic.

The electronic structure for Ca_10_(Pt_4_As_8_)(Fe_2_As_2_)_5_ has been studied via angle resolved photoemission spectroscopy (ARPES) [9]. Generally, there is a hole pocket and an electron pocket around the zone center. There are several electron pockets around the zone corner, among which two are suggested to be contributed by the FeAs layer, and several are contributed by Pt_4_As_8_ layers. Thus, the Pt_4_As_8_ layer is suggested to be metallic. Furthermore, the d_xz_ and d_yz_ bands are not degenerate at the Brillouin zone center (*Γ* point) and there is only hole-like Fermi surface at the *Γ* point originated from d_xy_ orbitals, it may be caused by the interaction between the Pt_4_As_8_ and FeAs layers [9]. 

In this paper, we studied the electrical properties of Ca_10_(Pt_4_As_8_)(Fe_2_As_2_)_5_ along its c-axis in order to understand how the interaction between Pt_4_As_8_ and FeAs layers influences the physical properties and anisotropy in this system. In addition, thermal conductivity has not yet been reported as another approach in measuring transport properties, and thus we also provide more extensive measurements and discussion on this approach.

## 2. Material Preparation and Characterization Methods

The preparation process is similar to descriptions provided elsewhere [2,4]. To grow single crystals of Ca_10_(Pt_4_As_8_)(Fe_2−_*_x_*Pt*_x_*As_2_)_5_, stoichiometric amounts of high purity Ca shot (99.999%, Alfa Aesar, Haverhill, MA, USA), Fe powder (99.95%, Alfa Aesar, Haverhill, MA, USA), Pt powder (99.95%, Alfa Aesar, Haverhill, MA, USA), and As powder (99.999%, Alfa Aesar, Haverhill, MA, USA) were mixed in the ratio of 17:14:9:31. The mixture was placed in an Al_2_O_3_ crucible (Gongtao Ceramics, Shanghai, China) and sealed in a quartz tube (Gongtao Ceramics, Shanghai, China) under vacuum. The whole ampule was heated to 700 °C in a box furnace (Henan Sante, Luoyang, China) at a rate of 140 °C/h and kept at this temperature for 5 h. It was further heated to 1100 °C at a rate of 80 °C/h where it was held for 50 h and then cooled to 1050 °C at a rate of 1.25 °C/h. It was further cooled to 500 °C in the next 100 h and finally cooled down to room temperature naturally by switching the power off. The cooling process is crucial because the mixture melts when it heats up and forms in a stable phase when it cools down. Shiny plate-like single crystals were obtained with a typical size of 3 mm × 3 mm × 0.8 mm, and a photo of the as prepared sample is shown in Figure 1b. 

Crystal structure and phase purity were checked by both single-crystal and powder X-ray diffraction, which were carried out on a Rigaku-D/max X-ray diffractometer (XRD) (Rigaku, Tokyo, Japan) using Cu-Kα radiation (*λ* = 1.54056 Å). Powder X-ray diffraction employed the powder grounded from the as-grown single crystals. The crystal used for this experiment was selected from the same batch as that used for composition and physical properties measurements. The chemical composition of the sample was identified by Energy Dispersive X-Ray Spectroscopy (EDX) (Hitachi, Tokyo, Japan). Electrical resistivity, magnetoresistance, Hall effect, thermal conductivity, and Seebeck coefficient measurements were performed in a Quantum Design PPMS System. Both ab plane and c-axis resistivities were measured using the standard four-probe technique.

## 3. Results and Discussion

Figure 1c shows the X-ray diffraction spectrum for the as-grown thin, plate-like single crystal along the c-axis. Note that only (00*l*) peaks are observed and the pattern matches very well with that previously reported in [4]. A powder XRD spectrum is shown in Figure 1d. The result exhibits the peaks from diffractions not only (00*l*) planes but also (*hkl*) planes with nonzero *h*, *k*, *l*. X-ray structure determination confirms that the lattice parameters and angles for the sample are *a* = 8.7548(12) Å, *b* = 8.7642(10) Å, *c* = 10.69005(8) Å, and *α* = 94.674(9)°, *β* = 104.396(8)°, *γ* = 90.037(10)°, respectively, indicating our sample crystallizes in a triclinic structure with space group symmetry $P\bar{1}$ at room temperature. No other impurities such as FeAs and PtAs_2_ were observed in any X-ray spectrum.

**Figure 2:**
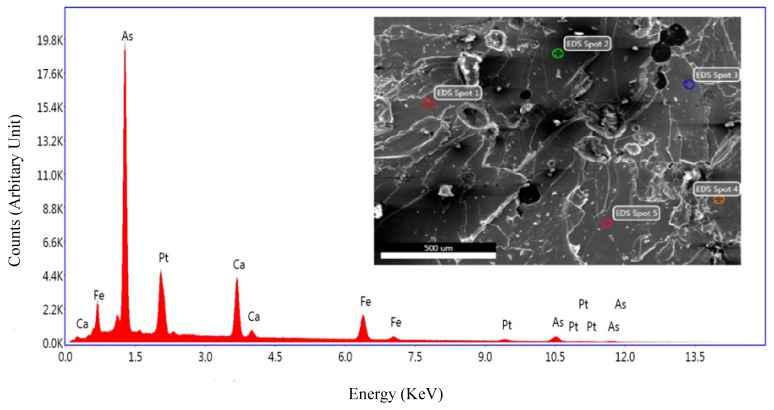
EDX measurement spectrum of Ca_10_Pt_5.4_Fe_8.6_As_18_; the inset figure is a surface SEM image of the measured sample.

The chemical composition was identified by the EDX measurement. A scanning electron microscope (SEM) image measured on the surface of the single crystal is shown in the Figure 2 inset. For each sample, five locations scattered on the surface were chosen for EDX measurement. An average of these scans was calculated. The results of the different samples are consistent with each other, implying homogenous growth was acquired throughout the batch. The average deviation is 3% for Ca, 5% for Pt, and 1% for Fe. The measured composition of the single crystals is Ca_10_Pt_5.4_Fe_8.6_As_18_, corresponding to a formula of Ca_10_(Pt_4_As_8_)((Fe_0.86_Pt_0.14_)_2_As_2_)_5_ if considering the Pt substitution effect. 

**Figure 3:**
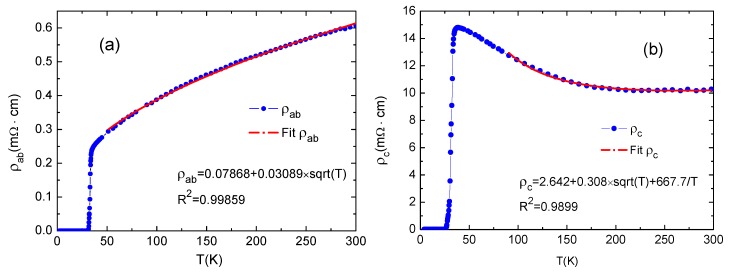
(**a**) The temperature dependence of the in-plane resistivity *ρ*_ab_ and (**b**) out-of-plane resistivity *ρ*_c_.

Figure 3 displays the temperature dependence of the in-plane (*ρ*_ab_) and out-of-plane resistivity (*ρ*_c_) of Ca_10_(Pt_4_As_8_)((Fe_0.86_Pt_0.14_)_2_As_2_)_5_ single crystals between 2 K and 300 K. The magnitude of *ρ*_ab_ and its overall features are similar to the previous reports [2]. The normal-state *ρ*_ab_ decreases with decreasing *T* ($\frac{d\rho_{\mathrm{ab}}}{dT}>0$), showing a metallic behavior. It drops sharply at *T*_c_onset_ = ~34 K, reaching zero-resistivity state at 31.2 K. The transition width in temperature is less than 3 K, indicating that our sample has high quality and spatial homogeneity. The residual resistivity ratio (RRR) (300K)/*ρ*(*T*_c_onset_) is 2.4. Relatively large *T*_c_onset_ and small RRR reflects the presence of Pt doping on the Fe site in FeAs layers as suggested in [1,2,3]. However, the out-of-plane resistivity *ρ*_c_ behaves strikingly different with *ρ*_ab_; it exhibits nearly independent *T* at higher temperatures and starts to increase with decreasing *T* ($\frac{d\rho_{c}}{dT}<0$) below 200K, showing a nonmetallic behavior. It then goes through a sharp peak around 38 K before dropping to zero. The anisotropic property between *ρ*_ab_ and *ρ*_c_ has not been noted in previous reports [1,2,4]. We acknowledge that this *ρ*_c_–*T* profile is reproducible and it should be an intrinsic property of Ca_10_(Pt_4_As_8_)(Fe_2−*x*_Pt*_x_*As_2_)_5_.

**Figure 4:**
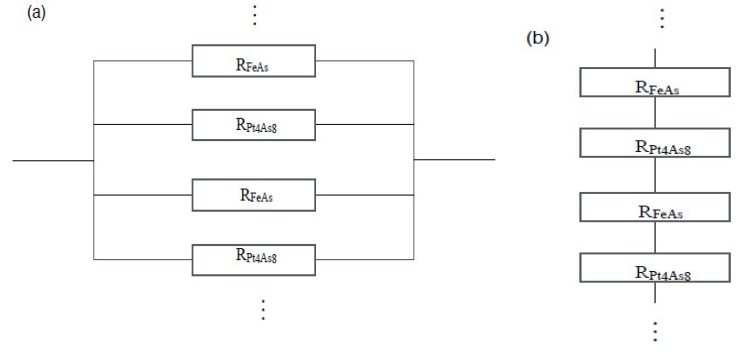
(**a**) A schematic plot of resistors made of FeAs layers and Pt_4_As_8_ layers connected in parallel; (**b**) a schematic plot of resistors made of FeAs layers and Pt_4_As_8_ layers connected in series.

Due to the layered crystal structure of Ca_10_(Pt_4_As_8_)((Fe_0.86_Pt_0.14_)_2_As_2_)_5_, the in-plane resistivity may be understood as a net resistivity of parallel connected resistors. A schematic of such a system is shown in Figure 4a. Here, we do not consider the resistance from the Ca layer because the FeAs and Pt_4_As_8_ layers are the main contributors of the electronic structure around the Fermi surface [9]. The net resistance (R) can be written as:

(1) $$R=1/\left[n\left(\frac{1}{R_{FeAs}}+\frac{1}{R_{Pt_{4}As_{8}}}\right)\right]$$

in which *n* is the number of Pt_4_As_8_–FeAs layers, and R_FeAs_ and R_Pt_4_As_8__ are the resistance of the FeAs layer and Pt_4_As_8_ layer, respectively. If the Pt_4_As_8_ layer is semiconducting or insulating, it should have a much larger resistance than the FeAs layer. Then, the net resistance R should be dominated by the resistance of the Pt_4_As_8_ layer and exhibit nonmetallic behavior. Yet, according to our results, the normal state *ρ*_ab_ shows metallic behavior, indicating the spacer layer Pt_4_As_8_ is metallic, which agrees with previous reports [2,4,9].

The normal state *ρ*_ab_ of some traditional FeAs-based superconductors shows metallic behavior and may have linear dependence on temperature due to the inelastic scattering originating from electron-phonon interactions. In some cuprates, *ρ*_ab_ has *T*^1.5^ dependence, implying the Fermi-liquid behavior. Yet, the temperature dependence of the normal state *ρ*_ab_ of Ca_10_(Pt_4_As_8_)((Fe_0.86_Pt_0.14_)_2_As_2_)_5_ is neither linear, quadratic temperature dependent, nor *T*^1.5^ dependent, as observed in cuprates or other FeAs-based compounds [10]. The normal state *ρ*_ab_ from 50 K to 300 K can be well fitted by $\rho_{ab}=A+B\sqrt{T}$ with *A* = 0.079 ± 0.008 mΩ cm and *B* = 0.031 ± 0.003 mΩ cm K^−1/2^. The red solid line in Figure 3a is the fit to the experimental data of the normal state *ρ*_ab_. All reported *ρ*_ab_ for Ca_10_Pt_4_As_8_(Fe_2_As_2_)_5_ has the similar square-root temperature dependence regardless of *T*_c_ [1,2,4], and thus, it may be an intrinsic property to Ca_10_Pt_4_As_8_(Fe_2_As_2_)_5_.

The square-root temperature dependence of electrical resistivity is normally expected at low temperatures in disordered metals and degenerate semiconductors because of interference with scattering by impurities [11,12]. The studies on the Fermi surface of Ca_10_Pt_4_As_8_(Fe_2_As_2_)_5_ imply that the Pt_4_As_8_ layer contributes several electron pockets. Thus, there may be competition between the Pt_4_As_8_ layer and the negatively-charged FeAs layer for electrons, leading to possible Pt deficiency in the Pt_4_As_8_ layer and partial substitution of Fe by Pt in the Fe_1−*x*_Pt*_x_*As layer [3,5,13,14]. As a result, charge carriers in both the Pt_4_As_8_ and Fe_1−*x*_Pt*_x_*As layers could experience the effect of disorder, which may be the reason why the in-plane electrical resistivity has the square-root temperature dependence. 

As shown in Figure 3b, *ρ*_c_ is much larger than *ρ*_ab_ in the normal state. It can be understood with a schematic plot of the net resistance R = *n* (*R*_FeAs_ + *R*_Pt_4_As_8__) along the out-of-plane direction shown in Figure 4b. Different from the normal state *ρ*_c_ of some cuprates which can be described using the equation *ρ*_c_ = A/T + B × T exhibiting the non-Fermi liquid behavior, the nonmetallic normal state *ρ*_c_ of Ca_10_(Pt_4_As_8_)((Fe_0.86_Pt_0.14_)_2_As_2_)_5_ can be appropriately fitted using the equation $\rho_{c}=A^{\prime }+B^{\prime }\sqrt{T}+C^{\prime }T^{-1}$. The solid line in Figure 3b shows the fit of *ρ*_c_ from 90 K to 300 K. The fitting parameters are calculated as follows: *A*′ = 2.642 ± 0.009 mΩ cm, *B*′ = 0.308 ± 0.006 mΩ cm K^−1/2^, and *C*′ = 667.7 ± 15 mΩ cm K.

The square-root temperature dependent component in *ρ*_c_ is due to the in-plane scattering, which may be intrinsic as Anderson proposed for high-*T*_c_ cuprates [15] or extrinsic due to a possible stacking fault [2]. The *T*^−1^ term can be attributed to interlayer incoherent scattering, similar to the properties of high-*T*_c_ cuprates [15] and (Sr_4_V_2_O_6_)Fe_2_As_2_ [16]. It implies the spacer layer Ca_10_Pt_4_As_8_ is not superconducting in nature, so Josephson junctions are formed along the c-axis of Ca_10_Pt_4_As_8_(Fe_2_As_2_)_5_ by stacking of the FeAs superconducting layer, the Ca_10_Pt_4_As_8_ semiconducting layer, and the FeAs superconducting layer. 

**Figure 5:**
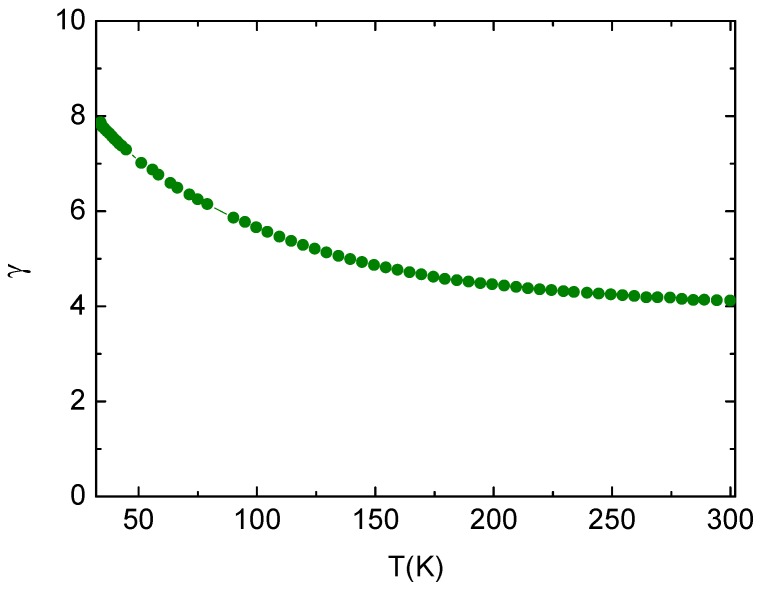
The temperature dependence of normal state anisotropy $\gamma =\sqrt{\rho_{c}/\rho_{ab}}$.

The resistivity anisotropy *γ* is calculated by $\gamma =\sqrt{\rho_{c}/\rho_{ab}}$ and it increases from ~4.1 at 300 K to ~7.9 around *T*_c_ as displayed in Figure 5. The *γ* of Ca_10_Pt_4_As_8_(Fe_2_As_2_)_5_ is larger than that of LaFeAsO_0.9_F_0.1_ [17]. Relatively large anisotropy and a conspicuous difference in the behavior of *ρ*_c_ and *ρ*_ab_ suggests rgw strong 2D nature of electronic structures in Ca_10_Pt_4_As_8_(Fe_2_As_2_)_5_, which is rarely encountered in traditional Fe-based superconductors. 

**Figure 6:**
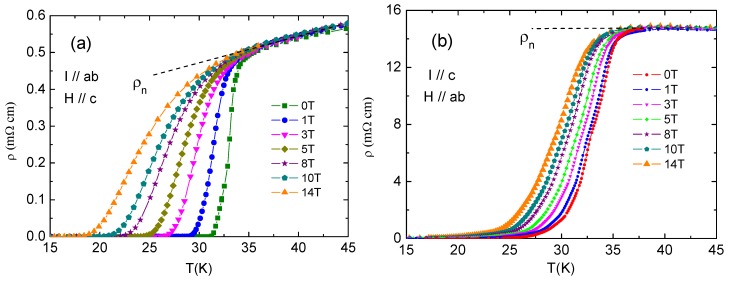
(**a**) The temperature dependence of *ρ*_ab_ with applied magnetic field *H* = 0, 1, 3, 5, 8, 10, and 14 T along the c direction in the superconducting state; (**b**) the temperature dependence of *ρ*_c_ with applied magnetic field *H* = 0, 1, 3, 5, 8, 10, and 14 T along the ab plane in the superconducting state.

The resistivity anisotropy (*γ*) in the superconducting state of Ca_10_(Pt_4_As_8_)((Fe_0.86_Pt_0.14_)_2_As_2_)_5_ was evaluated through resistivity measurements with an applied magnetic field *H*. Figure 6 shows the temperature dependence of *ρ*_ab_ and *ρ*_c_ under *H* along both the c-axis and the ab plane. 

In the case of *I*//ab and *H*//c-axis, it can be seen that the zero resistivity transition temperature (*T*_c0_) decreases with the rising applied magnetic field accompanied by an increase in the transition width. The *T*_c0_ is 18 K and the transition width *∆T*_c_ is 16 K for *H* = 14 T, considering *T*_c_onset_ is 34 K. This type of superconducting transition broadening with increasing magnetic field (*H*) is rarely seen in Fe-based superconductors but very common in cuprates due to the presence of strong thermal vortices fluctuations [18]. 

Interestingly, the superconducting transition width *∆T*_c_ does not change with an applied field when *I*//c and *H*//ab plane (Figure 6b) and *H* pushes both *T*_c_onset_ and *T*_c0_ to the lower temperature by the same amount. This shift is also much smaller than that of *∆T*_c_ for cases *I*//ab and *H*//c. 

**Figure 7:**
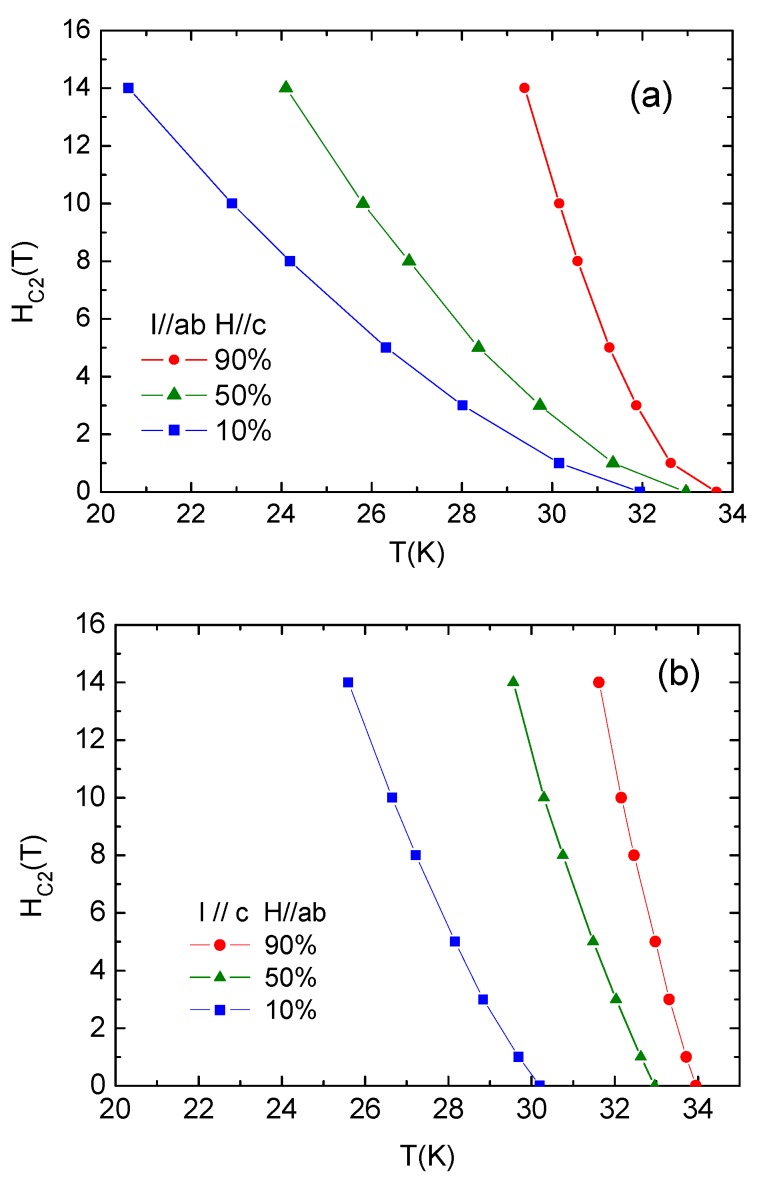
Temperature dependence of the upper critical field *H_c_*_2_(*T*) with (**a**) *H*//c and(**b**) *H*//ab.

As shown in Figure 7, the *H_c_*_2_ (*T*) of Ca_10_(Pt_4_As_8_)((Fe_0.86_Pt_0.14_)_2_As_2_)_5_ was extracted by identifying the *T*_c_(*H*) at which resistivity drops to 90% *ρ*_n_, 50% *ρ*_n_, and 10% *ρ*_n_. *ρ*_n_ is the normal resistivity before superconducting transition, as indicated by dashed lines in Figure 6.

**Figure 8:**
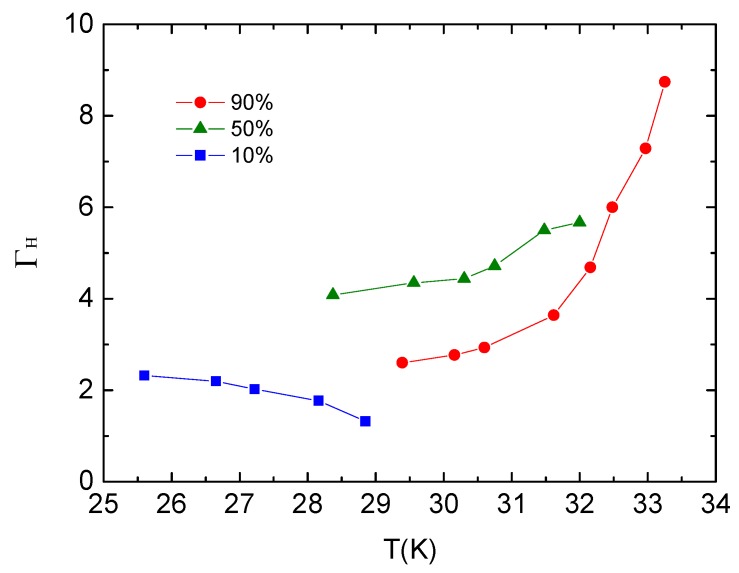
The temperature dependence of the *H*_c2_ anisotropy parameter $\Gamma_{H}=\frac{H_{c2}^{ab}}{H_{c2}^{c}}$.

The temperature dependence of the *H*_c2_ anisotropy parameter $\Gamma_{H}=\frac{H_{c2}^{ab}}{H_{c2}^{c}}$ obtained from Figure 7 is presented in Figure 8. *Γ*_H_ taken at 90% resistivity drop reaches 8 near *T*_c_, which is very close to the normal-state *γ* we obtained previously in the resistivity measurements. *Γ*_H_ varies from 4 to 6 using a 50% criteria as compared to 122 type. For example, with *Γ*_H_ = ~2 for BaFe_2_As_2_ [19,20], the *H*_c2_ anisotropy of Ca_10_(Pt_4_As_8_)((Fe_0.86_Pt_0.14_)_2_As_2_)_5_ is much larger.

It also can be noted from Figure 7b that $H_{c2}^{ab}\left(T\right)$ increases almost linearly with decreasing temperature, while $H_{c2}^{c}\left(T\right)$ exhibits an upward shape with a steep increase at low temperatures. This phenomena quite possibly originates from the multi-band effect such as in the NdFeAsO_0.7_F_0.3_ and MgB_2_ systems [21,22]. Thus, the positive curvature of $H_{c2}^{c}\left(T\right)$ reflects a multi-band nature in electrical structure for Ca_10_(Pt_4_As_8_)(Fe_2_As_2_)_5_.

Upper critical field *H_c_*_2_ is an important parameter for all superconductors especially in their practical applications. We estimated *H*_c2_ at 0 K using the Werthamer–Helfand–Hohenberg (WHH) approximation [23]:

(2) $$H_{c2}\left(0\right)=-0.69T_{c}\times {\left|\frac{dH_{c2}}{dT}\right|}_{T_{c}}$$

The corresponding coherence lengths are calculated via the Ginburg Landau (GL) formula:

(3) $$\{\begin{array}{c} \xi_{\mathrm{ab}}\left(0\right)=\sqrt{\frac{\varphi_{0}}{2\pi H_{c2}^{c}\left(0\right)}}\\ \xi_{c}\left(0\right)=\frac{\varphi_{0}}{2{\pi \xi }_{\mathrm{ab}}H_{c2}^{ab}\left(0\right)} \end{array}$$

where $\varphi_{0}=2.07\times 10^{-15}$ *Wb*. The obtained results are listed in Table 1. Although all derived values depend on how *H*_c2_(*T*) is extracted, the large difference of $H_{c2}^{ab}$ and $H_{c2}^{c}$ implies Ca_10_(Pt_4_As_8_)((Fe_0.86_Pt_0.14_)_2_As_2_)_5_ exhibits a large anisotropy in its superconducting state. Furthermore, the values of *ξ_c_*(0) derived from different criteria are all less than 10.69005(8) Å, i.e., the length of c-axis of the unit cell, suggesting that Ca_10_(Pt_4_As_8_)((Fe_0.86_Pt_0.14_)_2_As_2_)_5_ meets the dimensional requirement of fabricating the intrinsic Josephson junctions (iJJs).

**Figure 9:**
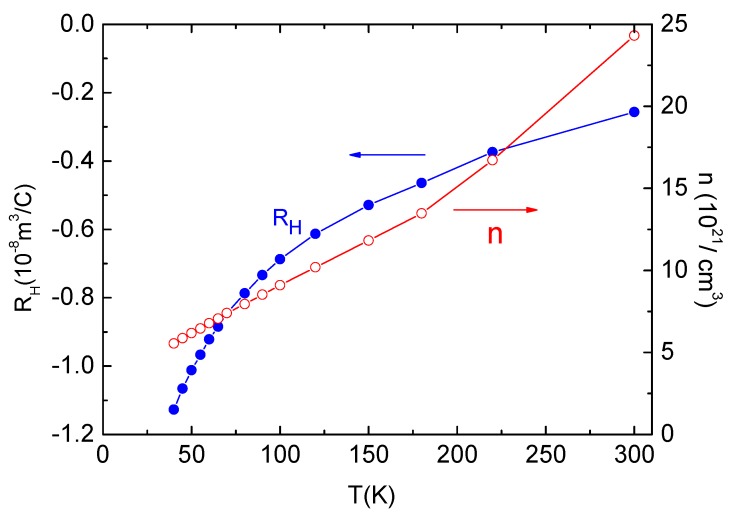
Hall coefficients *R*_H_(*T*) (blue dots) and carrier concentration (red circles) between 40 K and 300 K for Ca_10_(Pt_4_As_8_)((Fe_0.86_Pt_0.14_)_2_As_2_)_5_. The solid lines are guides for the eye.

For a normal metal with Fermi liquid behavior, the Hall coefficient (*R*_H_) is independent of temperature. The situation is more complex if the material has multi-band or non-Fermi liquid behavior such as, for example, cuprates or heavy fermions. Their Hall coefficient exhibits strong temperature and doping dependencies. The $H_{c2}^{c}\left(T\right)$ curves show the multi-band nature of Ca_10_(Pt_4_As_8_)((Fe_0.86_Pt_0.14_)_2_As_2_)_5_, so the Hall effect was measured by applying a magnetic field *H*⊥*I*//ab, and the data is presented in Figure 9. 

The transverse *R*_H_ remains negative at all temperatures above *T*_c_, indicating that the charge carrier is dominated by electrons. The magnitude of *R*_H_ increases with decreasing temperature, suggesting a multi-band and non-Fermi liquid behavior. Even though the doping in the FeAs layers is considered to be isovalent (i.e., Pt^2+^ replaces Fe^2+^) [2,5], it may still influence *R*_H_. Our *R*_H_(*T*) is similar with previous reports [1,2], but it exhibits stronger temperature dependence, which suggests that the carrier concentration cannot be solely determined by the Pt concentration in this material.

If a single band model is adopted, the corresponding carrier concentration (*n*) could be calculated with *R*_H_ via *n* = −1/eR_H_. The temperature dependence of *n* is shown as the red line in Figure 9. Generally, *n* decreases monotonically with decreasing *T*. The calculated *n* is about 2.5 × 10^22^ cm^−3^ at 300 K. It is comparable to other FeAs-based superconductors; for example, *n* of SrFe_2_As_2_ is about 1.52 × 10^22^ cm^−3^ at 300 K [24]. The carrier concentration indicates that the normal state of Ca_10_(Pt_4_As_8_)((Fe_0.86_Pt_0.14_)_2_As_2_)_5_ behaves as a good metal. Before superconducting transition, the carrier concentration drops to 0.5 × 10^22^ cm^−3^; however, it is still an order of magnitude larger than that of Ca10-3-8 (~10^21^ between 2 K and 300 K) [2]. This is because the one extra Pt atom in the Pt_4_As_8_ intermediary layer exceeds the Zintl valence satisfaction requirement and introduces redundant electrons into the system, leading to enhanced metallicity and a relatively higher *T*_c_.

**Figure 10:**
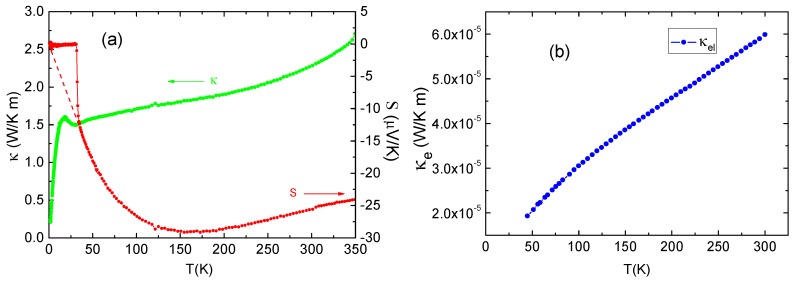
(**a**) Temperature dependence of thermal conductivity and the Seebeck coefficient for Ca_10_(Pt_4_As_8_)((Fe_0.86_Pt_0.14_)_2_As_2_)_5_; (**b**) electronic contribution to thermal conductivity above transition temperature *T_c_* evaluated by the Wiedemann–Franz law.

A different method of probing conduction mechanism is provided by thermal conductivity. We measured the temperature dependence of thermal conductivity and the Seebeck coefficient for Ca_10_(Pt_4_As_8_)((Fe_0.86_Pt_0.14_)_2_As_2_)_5_ from 2 K to 350 K, and the results are shown in Figure 10a.

The Seebeck coefficient (*S*) is negative at the measured temperature range above *T*_c_, with a value of −25.5604 μV/K at 300 K and a minimum value of −29.1343 μV/K near 154 K resulting from a phonon-drag contribution. This confirms that the electron-type charge carrier dominates in Ca_10_(Pt_4_As_8_)((Fe_0.86_Pt_0.14_)_2_As_2_)_5_, which is consistent with the Hall effect measurement. Then, *S* starts to increase with decreasing temperature and reaches zero steeply at the superconducting transition temperature. In addition, the Seebeck curve shows no anomalous enhancements associated with the crystal structure or spin density wave (SDW) transitions widely observed in undoped compounds such as SmFeAsO and BaFe_2_As_2_ [25,26], suggesting that there were no corresponding transitions in our sample at the temperatures measured.

With regard to the thermal conductivity (*κ*), it drops monotonously above *T*_c_ when temperature is lower than *T*_c_, *κ* and decreases sharply with the opening of the superconducting gap. Before the drastic drop, *κ* first displays an abrupt increase with a bump feature upon entering the superconducting (SC) state, and a similar behavior is commonly observed in the cuprate superconductors, e.g., YBa_2_Cu_3_O_7_ [27] and Bi_2_Sr_2_CaCu_2_O_8_ [28]. The enhancement of *κ* below *T*_c_ reflects the increase of the phonon mean free path by the condensation of charge carriers. Phonons then cease to dissipate their momentum in collisions with such a condensate.

The electronic contribution to thermal conductivity ($\kappa_{e}$) above *T*_c_ could be evaluated by the Wiedemann–Franz law $\kappa_{el}=\sigma LT$, where *σ* is the electrical conductivity of a metal and *T* is the temperature, *L*, known as the Lorenz number, which is equal to:

(4) $$L=\frac{\pi^{2}}{3}{\left(\frac{k_{B}}{e}\right)}^{2}=2.44\times 10^{-8}\;{W\Omega K}^{-2}$$

in which e is elementary charge and k_B_ is the Boltzmann constant. The calculated results are presented in Figure 10b. $\kappa_{el}$ is smaller by about 5 orders of magnitude than *κ* as shown in Figure 10a. Thus, heat in Ca_10_(Pt_4_As_8_)((Fe_0.86_Pt_0.14_)_2_As_2_)_5_ is mainly carried by phonons, and the electron contribution can be negligible. This is wholly different from the copper oxide superconductors. Ca_10_(Pt_4_As_8_)((Fe_0.86_Pt_0.14_)_2_As_2_)_5_ is a relatively low-charge carrier density system. 

It is worth noting that the *κ* value for Ca_10_(Pt_4_As_8_)((Fe_0.86_Pt_0.14_)_2_As_2_)_5_ is smaller than that for LaFeAsO_0.89_F_0.11_ [29]. We know that the main scattering mechanisms for phonons in crystal are carriers and structural defects, and intrinsic phonon–phonon scattering only exists in clean materials. While in the Ca_10_(Pt_4_As_8_)(Fe_2_As_2_)_5_ system, both the off-centered Pt atoms in the Pt_4_As_8_ plane and the substitutional Pt atoms in the FeAs plane introduce disorder into the crystal, which causes phonons to be strongly scattered and the crystal lattice vibration to localize, resulting in the rather smaller thermal conductivity of Ca_10_(Pt_4_As_8_)((Fe_0.86_Pt_0.14_)_2_As_2_)_5_.

## 4. Conclusions

In conclusion, X-ray diffraction, resistivity, Hall effect, Seebeck coefficient, and thermal conductivity measurements were performed on high quality Ca_10_(Pt_4_As_8_)((Fe_0.86_Pt_0.14_)_2_As_2_)_5_ single crystals. We observed metallic in-plane resistivity but non-metallic out-of-plane resistivity for Ca_10_Pt_4_As_8_(Fe_2_As_2_)_5_. The anisotropic property is unusual, and its normal state resistivity exhibits a large anisotropy (~8) near *T*_c_, making it one of the most anisotropic FeAs-based superconductors. The normal state in-plane resistivity has a square-root temperature dependence which is intrinsic to Ca_10_Pt_4_As_8_(Fe_2_As_2_)_5_. The interlayer incoherent scattering contributes to the out-of-plane transport property. A large coherence length along the ab plane and upper critical field were observed. Disorder and low-charge carrier density in the crystal may account for the relatively small thermal conductivity. The layered structure and the relatively higher transition temperature with the large electrical transport anisotropy of Ca_10_Pt_4_As_8_(Fe_2_As_2_)_5_ implies it may be a new good candidate for and have potential application in the fabrication of high frequency microelectronic devices such as next generation intrinsic Josephson junctions (IJJs).

## Figures and Tables

**Table 1:** The upper critical field and coherence length of Ca_10_(Pt_4_As_8_)((Fe_0.86_Pt_0.14_)_2_As_2_)_5_.

Criteria in Determining *H*_c2_	$\frac{dH_{c2}^{c}}{dT}T_{C}\left(T/K\right)$	$H_{c2}^{c}\left(0\right)$ (Tesla) (WHH Approach)	$\frac{dH_{c2}^{ab}}{dT}T_{C}\left(T/K\right)$	$H_{c2}^{ab}\left(0\right)$ (Tesla) (WHH Approach)	*ξ_ab_*(0) (Å)	*ξ_c_*(0) (Å)
0.9 *ρ*_n_	−6.5 ± 0.2	138 ± 5	−14.1 ± 0.1	313 ± 6	15.5 ± 0.2	6.8 ± 0.3
0.5 *ρ*_n_	−0.89 ± 0.1	19.7 ± 2	−4.4 ± 0.1	99 ± 2	40.9 ± 0.5	8.1 ± 0.3
0.1 *ρ*_n_	−0.74 ± 0.1	16.4 ± 1	−3.8 ± 0.1	84 ± 3	44.8 ± 0.4	8.7 ± 0.5

WHH: Werthamer–Helfand–Hohenberg.
